# Cathodal Transcranial Direct Current Stimulation of the Occipital cortex in Episodic Migraine: A Randomized Sham-Controlled Crossover Study

**DOI:** 10.3390/jcm9010060

**Published:** 2019-12-26

**Authors:** Rechdi Ahdab, Anthony G. Mansour, Georges Khazen, Christelle El-Khoury, Toni M. Sabbouh, Maher Salem, Wissam Yamak, Samar S. Ayache, Naji Riachi

**Affiliations:** 1 Division of Neurology, Lebanese American University Medical Center Rizk Hospital, Beirut 113288, Lebanon; chadahdab@gmail.com (R.A.); wissam.yamak@lau.edu.lb (W.Y.); 2 Division of Neurology, Gilbert and Rose Mary Chagoury School of Medicine, Lebanese American University, Byblos 4504, Lebanon; anthony.gmansour@gmail.com (A.G.M.); gkhazen@lau.edu.lb (G.K.); christelle.elkhoury@lau.edu (C.E.-K.); Maher.Salem@lau.edu (M.S.); 3 Division of Neurology, Hamidy Medical center, Tripoli 1300, Lebanon; 4 Department of Internal Medicine, Ohio State University, Columbus, OH 43210, USA; 5 Computer Science and Mathematics Department, Lebanese American University, Byblos 4504, Lebanon; 6 Division of Family Medicine, Lebanese American University Medical Center Rizk Hospital, Beirut 113288, Lebanon; 7 Department of Internal Medicine, Morristown Medical Center, Morristown, NJ 07960, USA; toni.sabbouh@lau.edu; 8 Service de Physiologie-Explorations Fonctionnelles, Hôpital Henri Mondor, Assistance Publique- Hôpitaux de Paris, 94010 Créteil, France; samarayache@gmail.com; 9 EA 4391, Excitabilité Nerveuse et Thérapeutique, Université Paris-Est-Créteil, 94000 Créteil, France

**Keywords:** cathodal tDCS, migraine, occipital cortex, pain, tablets

## Abstract

Summary: Three consecutive daily sessions of cathodal transcranial direct current stimulation (tDCS) was sufficient to show a significant decrease in headache duration and intensity as well as tablets consumption, in patients suffering from episodic migraine. Background: Migraine prophylaxis is recommended in patients with frequent and/or intense headaches, but poor tolerability and lack of efficacy of preventive drugs are common in clinical practice. Hence, new prophylactic strategies are needed. Objective: The aim of this study was to evaluate the efficacy of tDCS in terms of migraine prophylaxis. Methods: This was a double blind and sham-controlled trial. Forty-two migraine patients were randomly assigned in a crossover design to receive three consecutive daily sessions of both sham and cathodal tDCS stimulation (2.0 mA, 20 min) over the occipital cortex of the dominant side of the migraine pain (O1/O2). Migraine duration and intensity, number of analgesic tablets, and number of headache-free days (where no headache abortive medications are taken) were recorded one week before and two weeks after treatment. A washout period of one week was allowed before crossing to the other treatment arm. Results: Relative to sham, cathodal stimulation was associated with a significant reduction in the number of headache days, tablets consumption, and pain intensity; and a significant increase in the number of headache-free days. These beneficial effects were sustained over two weeks. No serious side effects were observed, and the procedure was well tolerated. Conclusion: Based on these findings, cathodal tDCS applied to the occipital cortex seems to be an effective and well tolerated alternative to pharmacotherapy in patients with episodic migraine.

## 1. Introduction

Migraine is a complex neurological condition characterized by recurrent episodes of throbbing headaches of at least moderate intensity [1]. With an estimated prevalence of 13–18%, it is a leading cause of disease-related disability worldwide, surpassing that of stroke, epilepsy, and head trauma [2].

Management of migraine is based on pharmacological agents and avoidance of known migraine triggers. Migraine prevention is based on a number of drug classes including beta-blockers, antidepressants, and anti-epileptics, among others [3]. Epidemiological data have shown that only a subset of patients with frequent migraine attacks are offered prophylactic drugs [4], half of whom eventually discontinue treatment because of lack of efficacy or intolerable side effects [3]. As such, there is a great unmet need for novel treatments that are both effective and tolerable.

Various non-pharmacological treatments, such as vagal nerve stimulation, occipital nerve stimulation, and transcranial magnetic stimulation, have been tested in episodic migraine with variable results [5,6]. Transcranial direct current stimulation (tDCS) is a safe, practical, and widely available method to modulate cortical excitability [7,8]. It has been suggested that tDCS is effective in preventing migraine attacks based on its ability to correct abnormal cortical excitability [9,10,11,12,13,14,15]. We conducted a randomized, double-blind, sham-controlled study, with an active three-day intervention phase, to investigate the efficacy and side effects profile of tDCS as a preventive treatment of episodic migraine.

## 2. Materials and Methods

The study protocol was approved by our local institutional review board (IRB) (Hamidy Medical Center IRB, approval number HMC#5017, date of approval—21 January 2017) and was in accordance with the 1964 Declaration of Helsinki and its later amendments. All participants provided written informed consent prior to enrollment.

### 2.1. Study Population

Patients were recruited at the Hamidy Medical Center, a primary care multispecialty clinic in North Lebanon. Inclusion criteria included age between 18 and 60 years, history of episodic migraine with or without aura according to the International Headache Society (HIS) 3rd edition criteria, and at least 4 migraine days per month. Patients who were on migraine preventive medication were allowed to participate in the study if no changes in dosage had been implemented in the previous 3 months and none were anticipated in the following 2 months. Patients with chronic migraine, defined as greater than 15 headache days per month for three consecutive months, were excluded. Other key exclusion criteria were substance abuse, neuropsychiatric disorders, history of seizures, breast feeding, lactation, brain surgery, cardiac pacemakers, other chronic pain disorders, prior experience with tDCS, metallic hardware in the head or scalp (e.g., surgical clips), known brain metastasis, or the use of botulinum toxin in the head and neck period during the 6 months prior to screening.

### 2.2. Study Design

**Figure 1:**
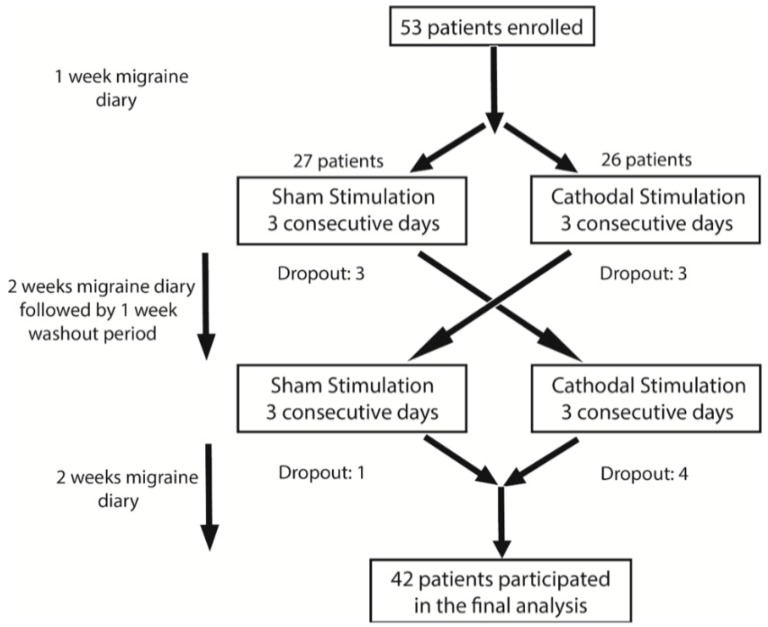
Schematic diagram summarizing the three phases of the study.

This is a randomized, double blind, sham-controlled, crossover trial including two treatment arms: cathodal and sham stimulation of the occipital cortex. The study design is summarized in Figure 1. It consisted of a screening visit, a 7-day pre-interventional period, a 3-day interventional period, and a 14-day post-interventional period. Eligible patients were randomly assigned to one of the two arms—active or sham tDCS stimulation. After completion of the post-interventional period, all subjects had a washout period of 1 week before crossing to the other arm. Patients and investigators analyzing the data were blinded to the assigned arm.

Participants were seen at five predefined visits. During the first visit (screening visit), patients were interviewed to determine whether their headaches qualify as migraines, and to check if they fulfill the inclusion and exclusion criteria. Eligible patients willing to participate in the study were then asked to sign the informed consent after the study protocol had been thoroughly explained. During this visit, participants were educated on how to fill in the migraine diary. The latter served to note the number of migraine days, and included a visual analogue scale (VAS) to evaluate intensity of migraine attacks, and entries to account for the number and type of headache tablets. Patients were asked to fill in their diaries, on a daily basis, for 7 days prior (baseline—pre-interventional period) and 14 days (week 1 and week 2—postinterventional period) following tDCS sessions. Patients were seen at the end of the pre- and post-interventional periods for both types of stimulation. The aim of these visits was to check for the completeness of the diary, hand in a new diary, address any specific concern, and record any lingering effect of tDCS.

Participants received two sets of three consecutive daily sessions of tDCS. The order in which the tDCS blocks were delivered (active followed by sham and vice versa) was randomly defined based on a 1:1 ratio.

### 2.3. Study Endpoints and Output Measures

The primary endpoint of this study was to explore the effect of cathodal occipital tDCS on the number of migraine days. The secondary endpoints were to evaluate the impact of this intervention on (i) the intensity of migraine attacks as per the VAS, (ii) the consumption of migraine medication, and (iii) the number of headache-free days (corresponding to the days where no headache medications were consumed).

Therefore, four output measures were calculated before and after (at week 1 and week 2) each block (sham and active). These include the following: number of migraine days at baseline, week 1 and week 2; mean of migraine pain intensity (VAS) at baseline, week 1, and week 2 (i.e., VAS_baseline_, VAS_week1_, and VAS_week2_); number of migraine tablets/week at baseline, week 1, and week 2; and number of headache-free days/week at baseline, week 1, and week 2.

### 2.4. Transcranial Direct Current Stimulation

A trained nurse was responsible for delivering the tDCS sessions. Participants were seated in a comfortable chair during the whole duration of the stimulation. Direct current was transferred by a saline-soaked pair of sponge electrodes (35 cm^2^) and was delivered using a Sooma tDCS^TM^ device (Sooma Oy, Helsinki, Finland). The cathode was placed over the occipital cortex of the dominant side of the migraine pain (i.e., O1/O2 according to the 10–20 international EEG system), and the anode electrode was placed over the supraorbital area on the opposite side. A current of 2.0 mA intensity was applied for 20 min for cathodal stimulation. For the sham stimulation, the electrodes were placed in the same position, but the current automatically phased off after 20 s.

### 2.5. Statistical Analysis

#### 2.5.1. Sample Size Calculation

It is important to mention that the minimum sample size was computed using the power.t.test function in the R statistical program, while considering a paired difference of 1 or more in the VAS to be significant with an 80% power and an alpha of 5%. The standard deviation of paired VAS was assumed to be 2. The minimum number of pairs was found to be 34. Considering a 15% possible drop-out rate, the minimum number of patients needed was estimated at 40.

#### 2.5.2. Data Analysis

A per protocol analysis was conducted. Analysis was performed using the R statistical program. Descriptive statistics are presented as mean ± standard error of the mean (SEM). Shapiro test was used to check for normality and since not all measures were normally distributed, Wilcoxon test was used to assess the effect of each condition on the above-mentioned measures. The *p*-values were adjusted using the Bonferroni method, and a *p*-value < 0.05 was considered as significant.

Comparison of VAS_baseline_ and number of migraine days at baseline, before active and sham conditions, was also done, using Wilcoxon test, to ensure that the washout period was sufficient to eliminate the effect of tDCS.

The difference in all the measures before and after each block was computed as baseline—week 1 and baseline—week 2 for weeks 1 and 2, respectively.

## 3. Results

A total of 53 patients were included in the study in the period extending between January to July 2017. Baseline demographics and key clinical data are summarized in Table 1.

A total of four patients dropped out of the study after receiving the sham stimulation and seven after the active stimulation. The reason for drop out was incomplete migraine diaries (one in the sham group and four in the active group), the lack of perceived benefit (two in the sham group), and significant improvement of migraine (one in the sham group and three in the active group). There were no instances of withdrawal due to side effects. In total, 42 patients successfully completed the study.

No significant difference was found between first and second block, concerning VAS_baseline_ and number of migraine days at baseline (*p* = 0.938 and 0.556, respectively). This confirms the adequacy of the washout period.

**Figure 2:**
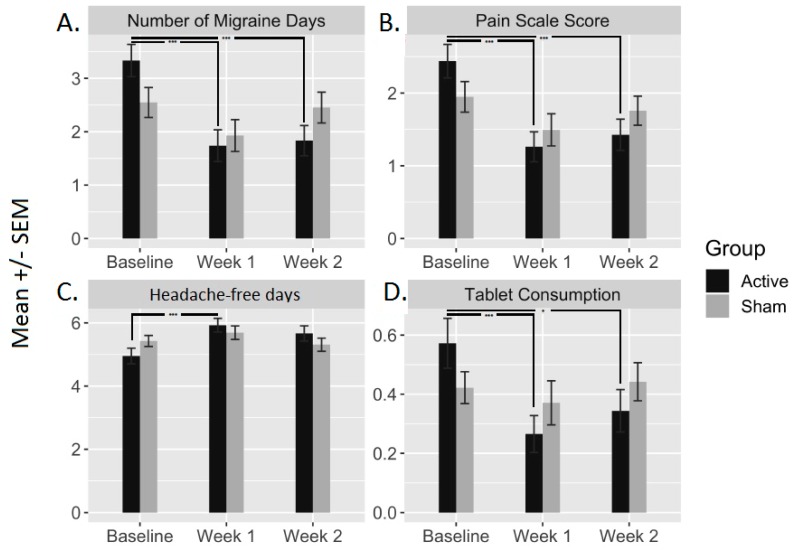
The bar plots represent the mean ± standard error of the mean (SEM) of the four output measures before (baseline) and after (week 1 and week 2) active and sham conditions. These output measures are (**A**) number of migraine days, (**B**) pain scale scores (VAS), (**C**) headache-free days and (**D**) tablets consumption. *** indicates a significance <0.001; * indicates a significance <0.05.

With respect to the primary endpoint, number of migraine days was significantly reduced following active tDCS. This reduction was observed at week 1 (1.60 (0.23), *p* < 0.001) and week 2 (1.50 (0.26), *p* < 0.001). Sham block did not result in significant changes (*p* > 0.05; Figure 2).

In addition, the active condition yielded a significant decrease in VAS scores (VAS_week1_: 1.17(0.17), *p* < 0.001; and VAS_week2_: 1.01 (0.19), *p* < 0.001)) and tablets consumption (at week 1: 0.30 (0.06), *p* < 0.001; and week 2: 0.22 (0.08), *p* = 0.002)), and a significant increase in the number of headache-free days (at week 1: (0.62 (0.11), *p* < 0.001; but not at week 2: 0.45 (0.14), *p* = 0.006)). No significant changes were observed following the sham block (Figure 2).

## 4. Safety and Tolerability

None of the patients terminated the stimulation, needed any medical intervention, or dropped out of the study because of adverse events. All patients completed the adverse effect questionnaire. All events were mild in severity, and they are summarized in Table 2.

## 5. Discussion

Our results showed a significant benefit of active cathodal occipital tDCS over sham stimulation, in terms of migraine prophylaxis in patients suffering from episodic migraine. This benefit translated clinically into a reduction in the number of migraine days (1.6 fewer migraine days per week) and migraine intensity, and a parallel reduction in tablets consumption. Treatment effects were observed as early as the first week and persisted for the entire follow-up period (two weeks in total). The immediate onset of action is in steep contrast with pharmacologic prevention of migraine that typically requires several weeks or months to start working [3,16]. Another advantage over preventive drugs is the excellent tolerability and the absence of serious side effects. Although patients were not followed up beyond two weeks, the beneficial effects of tDCS tended to wear off towards the end of the follow-up period and had completely disappeared after four weeks (the time the second baseline was measured).

The comparison of our results to existing data is limited by significant differences in study designs. Published studies showed some promising results regardless of the type of stimulation (anodal versus cathodal), the cortical target (primary motor cortex versus occipital cortex), and migraine subtype (episodic versus chronic). However, the level of evidence remains low owing to the many shortcomings of published trials. The latter were primarily pilot studies [9,10,13,14,15], included a small number of patients [9,13,14,15], or did not include any sham condition [15]. Two randomized double-blind trials compared cathodal tDCS to sham when applied to the occipital cortex [10,14]. Although both trials showed a trend towards a better outcome in the active tDCS group, comparison with the sham group showed no significant difference in terms of number of migraine attacks, duration of attacks, and intensity of pain [10,14]. These results are in contrast with our study, which clearly demonstrated a beneficial effect of cathodal tDCS that was apparent across all measured outcomes including the number of migraine attacks, pain intensity, and amount of migraine abortive treatments. The reason for this discrepancy is likely the difference in study design and sample size. The previous two studies were pilot studies and included a limited number of patients and were, therefore, probably underpowered to detect a difference between the two conditions (active and sham). Our study is larger than the above-mentioned trials and differs from them by having adopted a crossover design. The latter has the advantage of reducing the influence of confounding covariates since the patients serve as their own controls. Such a study design is particularly advantageous in episodic diseases such as migraine [17] and provides the best unbiased estimate for the difference between treatments.

Three other randomized controlled trials used the primary motor cortex as a target to treat episodic [11] and chronic migraine [9,13]. All of them showed beneficial effects of anodal tDCS when compared to sham in terms of number of migraine days [11,13] and quality of life [9]. In one study, Andrade and collaborators also targeted the left prefrontal cortex with anodal tDCS in subjects with refractory chronic migraine. They found a significant effect on quality of life in subjects with refractory chronic migraine that seemed to surpass that of primary motor cortex stimulation. More studies with direct comparison between the different targets are needed to determine the best stimulation protocol to prevent migraine attacks.

Although our results support the efficacy of tDCS in terms of migraine prevention, they provide no clue as to the underlying mechanisms of action. In particular, we did not investigate whether the observed benefits correlated with changes in cortical excitability. Subjects with migraine have been shown to have subtle functional and morphological abnormalities that manifest interictally [18]. These include abnormal visual processing [19,20], occipital cortex hypermetabolism as demonstrated by functional Magnetic Resonance Imaging (fMRI) [21,22], reduced cortical inhibition manifesting as low phosphene threshold [23], and reduced intracortical and cerebellar inhibition [24]. Since the occipital cortex is not classically considered an area involved in pain modulation and control, it is tempting to believe that the migraine preventive effects of tDCS is through correction of altered cortical excitability [25]. To the best of our knowledge, this has been addressed in only one study and no correlation was found between clinical improvement and changes in cortical excitability, as measured by the phosphene threshold [14]. Further studies are needed to provide better mechanistic insights on the antimigraine effects of tDCS. On the other hand, it is relevant to highlight that the anode in the present study was placed over the supraorbital region, facing the frontal lobe. As such, it can be argued that such supraorbital position could have led to current spreading to the left dorsolateral prefrontal cortex and thus could have played a role in the beneficial effects observed following active condition. It is difficult to exclude this possibility. Future studies using an extracephalic anodal site could help in clarifying this issue.

In conclusion, noninvasive neuromodulation using tDCS is a safe and well-tolerated treatment option for episodic migraine prevention. The technique is effective, widely available, and easy to implement. It has limited contraindications and carries no risk of medication-induced headaches. tDCS is particularly attractive in certain populations with limited therapeutic options such as pregnant/breastfeeding women, and patients with comorbidities and contraindications to medical treatment. Future studies are needed to determine the most appropriate maintenance dose for a sustained and durable effect.

## Figures and Tables

**Table 1:** Clinical and demographic characteristics of the participants. N: number of patients; NSAID: Non-steroidal anti-inflammatory drugs; OCP: Oral contraceptive pills.

Clinical and Demographic Characteristics	
Age (mean)	36.5
Age of Onset (mean)	23.7
Gender	*n*
Male	7
Female	35
Aura	*n*
Male with Aura	2
Male without Aura	5
Female with Aura	8
Female without Aura	27
Family History of Migraine	*n*
Present	30
Medication	*n*
NSAIDs	13
Paracetamol	23
Prophylactic Treatment	3
None	3
Using OCPs	3

**Table 2:** Percentage of patients reporting adverse effects of transcranial direct current stimulation (tDCS) during and directly after termination of stimulation blocks.

	During tDCS		After tDCS	
Adverse Effect	Active	Sham	Active	Sham
Headache	14	7	14	7
Pain	6.3	3.2	3.2	2.4
Fatigue	4.8	3.2	2.4	1.6
Disturbed Vision	4.8	1.6	2.4	0.8
Disturbed Concentration	4.0	1.6	3.2	0.8
Nervousness	1.6	0.8	0.0	0.0
